# Associations Between Canada's Cannabis Legalization and Emergency Department Presentations for Transient Cannabis-Induced Psychosis and Schizophrenia Conditions: Ontario and Alberta, 2015–2019

**DOI:** 10.1177/07067437211070650

**Published:** 2022-01-12

**Authors:** Russell C. Callaghan, Marcos Sanches, Robin M. Murray, Sarah Konefal, Bridget Maloney-Hall, Stephen J. Kish

**Affiliations:** 1 Northern Medical Program, 6727 University of Northern British Columbia (UNBC), Prince George, British Columbia, Canada; 2 School of Population and Public Health, University of British Columbia (UBC), Vancouver, British Columbia, Canada; 3 University of Victoria, Canadian Institute for Substance Use Research, Victoria, British Columbia, Canada; 4 Biostatistical Consulting Unit, Centre for Addiction and Mental Health, Toronto, Ontario, Canada; 5 Department of Psychosis Studies, Institute of Psychiatry, Psychology, and Neuroscience, King’s College, London, UK; 6 Canadian Centre on Substance Use and Addiction, Ottawa, Ontario, Canada; 7 Human Brain Laboratory, Centre for Addiction and Mental Health, Toronto, Ontario, Canada

**Keywords:** emergency psychiatry, psychosis, cannabinoids, medicolegal issues

## Abstract

**Objective:**

Cannabis legalization in many jurisdictions worldwide has raised concerns that such legislation might increase the burden of transient and persistent psychotic illnesses in society. Our study aimed to address this issue.

**Methods:**

Drawing upon emergency department (ED) presentations aggregated across Alberta and Ontario, Canada records (April 1, 2015–December 31, 2019), we employed Seasonal Autoregressive Integrated Moving Average (SARIMA) models to assess associations between Canada's cannabis legalization (via the *Cannabis Act* implemented on October 17, 2018) and weekly ED presentation counts of the following ICD-10-CA-defined target series of cannabis-induced psychosis (F12.5; *n* = 5832) and schizophrenia and related conditions (“schizophrenia”; F20-F29; *n* = 211,661), as well as two comparison series of amphetamine-induced psychosis (F15.5; *n* = 10,829) and alcohol-induced psychosis (F10.5; *n* = 1,884).

**Results:**

ED presentations for cannabis-induced psychosis doubled between April 2015 and December 2019. However, across all four SARIMA models, there was no evidence of significant step-function effects associated with cannabis legalization on post-legalization weekly ED counts of: (1) cannabis-induced psychosis [0.34 (95% CI −4.1; 4.8; *P* = 0.88)]; (2) schizophrenia [24.34 (95% CI −18.3; 67.0; *P* = 0.26)]; (3) alcohol-induced psychosis [0.61 (95% CI −0.6; 1.8; *P* = 0.31); or (4) amphetamine-induced psychosis [1.93 (95% CI −2.8; 6.7; *P* = 0.43)].

**Conclusion:**

Implementation of Canada's cannabis legalization framework was not associated with evidence of significant changes in cannabis-induced psychosis or schizophrenia ED presentations. Given the potentially idiosyncratic rollout of Canada's cannabis legalization, further research will be required to establish whether study results generalize to other settings.

## Introduction

On October 17, 2018, the Canadian Federal government implemented the *Cannabis Act*—the legislative framework for the legalization of non-medical cannabis use in Canada.^1^ The movement toward cannabis legalization in many jurisdictions worldwide has raised concerns that such legislation may increase the burden of transient and persistent psychotic syndromes in society.^2,3^ Prior experimental studies administering delta-9-tetra-hydrocannabinol (THC; the primary psychoactive component in cannabis) to healthy participants have provided evidence that THC administration can cause dose-dependent patterns of positive and negative psychotic symptomology in otherwise healthy individuals.^4,5^ In addition, a large body of epidemiological evidence (utilizing a range of research designs) has demonstrated a directional dose-response association between cannabis use and incidence of schizophrenia, with the association being more pronounced among individuals reporting early adolescent initiation, those with a high genetic loading for psychotic illnesses, and persons reporting prior psychotic symptoms.^6–9^ Experimental pharmacological challenge involving patients with schizophrenia has shown that administration of THC worsens psychotic symptoms (e.g., D'Souza et al. 2005),^10^ while clinical studies (for a systematic review, see, Schoeler et al. 2016)^11^ have found that the continuation of cannabis use after the onset of psychotic illness is associated with worse outcomes—including higher relapse rates, longer hospital stays, and more pronounced positive psychotic symptomology, in comparison to those patterns observed among people with psychosis who discontinue or abstain from cannabis use. It is important to acknowledge, however, that only a small proportion of cannabis users identified in population-based surveys report any past-year problems related to their cannabis use.^12^

Cannabis legalization might support increased patterns of cannabis use in society through a number of potential mechanisms, such as decreased perceived harmfulness, increased cannabis availability, elimination of criminal penalties as a deterrent to using, and changes in social norms making cannabis use more socially acceptable.^13,14^ Cannabis legalization may also lead to net societal benefits, such as decreased impacts of a cannabis-related burden on the justice system,^15^ reductions in cannabis-related criminalization and stigma, regulated and quality-controlled access to cannabis products, and development of cannabis-related treatment protocols and harm reduction interventions.^16,17^ In this context, we tentatively expected that cannabis legalization would increase the number of hazardous cannabis-use episodes leading to the experience of cannabis-related harms in the populations of Ontario and Alberta—a pattern which, in turn, would likely contribute to detectable increases in ED presentations for cannabis-induced psychosis and, perhaps, more persistent psychotic syndromes (i.e., schizophrenia and related conditions). The *Cannabis Act* mandates a Parliamentary review of the impacts of cannabis legalization on Canadian public health, and this study aims to provide evidence for that review.^1^

## Methods

The Centre for Addiction and Mental Health (CAMH) Research Ethics Board (REB) provided ethical approval for the current study (REB# 074/2019). The Canadian Institute for Health Information (CIHI) supplied de-identified data for the project, and patient consent was not required for the release of these data.

Data source: National Ambulatory Care Reporting System (NACRS), April 1, 2015–December 31, 2019.

The current study utilized data from all Ontario EDs (with ∼177 sites, receiving 6.4 million care episodes annually) and all Alberta EDs (with ∼107 sites, receiving 2.3 million care episodes annually), spanning from April 1, 2015–December 31, 2019, from the NACRS database (held and managed at CIHI). Alberta and Ontario are the only two Canadian provinces which submit 100% of all ED presentations in the province to the NACRS data system and, as a result, we selected only these two provinces for our current study. Most other provinces and territories submit only a subset of all of the ED presentations occurring in their province/territory to the NACRS database.^18^ The lead authors (RCC, MS) are bound by data-security and data-privacy agreements with CIHI. As a result, the research team cannot release the raw data from the study. Study data are publicly available from the data guardian (CIHI; snap@cihi.ca) upon request and successful application.

### Outcome Data Series

In the NACRS database, each ED medical record included at least one ICD-10-CA-based primary diagnosis,^19^ along with optional fields for nine supplemental diagnoses. The primary and secondary/comparison outcomes in the current study were defined as an occurrence, in any diagnostic position in a patient's emergency department medical record, of at least one of the identifying ICD-10-CA codes: F12.5 (cannabis-induced psychosis)^20,21^; F20-F29 (Schizophrenia and related disorders)^22^; F10.5 (alcohol-induced psychosis)^21^; or F15.5 (amphetamine-induced psychosis).^20,21^ Our ICD-10-based transient alcohol- or drug-induced psychosis and schizophrenia outcomes have been widely used in prior case-registry studies (e.g., ^21,22^). The amphetamine-psychosis and alcohol-psychosis outcomes were chosen to serve as drug-related comparison series to our primary cannabis-related psychosis series. We included all NACRS records with an identifying ICD-10 code; no exclusion criteria were applied. Counts of the primary and secondary outcomes were aggregated across weeks (equaling 7-day periods), centered at the date of Canadian cannabis legalization (October 17, 2018). All data series were aggregated across Ontario and Alberta ED presentations.

### Outcome Data Series: Samples

The data-series samples consisted of ED presentations occurring during the data span (April 1, 2015–December 31, 2019) in Alberta and in Ontario for (1) cannabis-induced psychosis [*n* = 5832; 77.1% male; mean age, 28.1 years (standard deviation, *sd* = 10.5)]; (2) schizophrenia and related conditions [*n* = 211,661; 63.2% male; mean age, 39.2 years (*sd* = 15.7)]; (3) amphetamine-related psychosis [*n* = 10,829; 66.9% male; mean age, 32.9 years (*sd* = 9.8)]; and (4) alcohol-related psychosis (*n* = 1884; 69.2% male; mean age, 37.7 years (*sd* = 13.7).

### Analytic Plan

Interrupted time series analysis was used to test separately the effects of cannabis legalization on primary and secondary data-series outcomes in Ontario and Alberta, following the framework developed by Box and colleagues.^23^ We employed Seasonal Autoregressive Integrated Moving Average (SARIMA) models using the framework of Cryer and Chan.^24^ Four primary SARIMA models were developed based on the following weekly ICD-10-CA-defined data series: (1) cannabis-induced psychosis; (2) schizophrenia and related conditions; (3) amphetamine-related psychosis; and (4) alcohol-related psychosis. The step format for the primary effect in each model was chosen initially because it is consistent with the definition of the actual legalization intervention (exposure), which remained throughout the post-intervention period.

In the first phase of the modeling, the 185-week pre-legalization series was used to identify SARIMA models in order to achieve stationarity in each model. In the current study, the seasonal lag term was 52 weeks. We identified suitable models to achieve stationarity by examining the autocorrelation and partial autocorrelation functions at different lags,^24^ comparing the Akaike Information Criterion values (AIC),^25^ and testing the residuals of the models using the Durbin-Watson test.^26^ The SARIMA models achieve stationarity with the following parameters, depicted in the SARIMA notation: (1) cannabis-induced psychosis, SARIMA [(0,1,2)(1,1,0)_52_]; (2) schizophrenia, SARIMA [(0,1,1)(1,1,0)_52_]; (3) amphetamine-related psychosis, SARIMA [(0,1,1)(1,1,0)_52_]; and (4) alcohol-related psychosis, SARIMA [(0,1,1)(1,1,0)_52_].

After reasonable models were found for the pre-legalization series, the full data series were used and step transfer functions were selected as the primary approach to estimate the potential step-function effects of cannabis legalization in the four data series.^24^ In simple terms, the pre-legalization SARIMA coefficients were used to forecast the predicted patterns of the outcomes across the post-legalization period, and these predicted patterns were compared to the observed patterns occurring after legalization in order to assess whether cannabis legalization might have affected the observed post-legalization data points. Subsequently, we also estimated more flexible SARIMA models by incorporating a parameter for a step function and a parameter capable of estimating the possible increase or attenuation of the initial step function.^24^ We tested for possible innovative outliers and additive outliers in models using the framework described in Cryer and Chan (see Section 11.2).^24^

All models were estimated in R 4.0.2^27^ using the package TSA 1.3.^28^ The study team conducted the statistical analyses from January-February, 2021. The primary research question and associated analyses were not pre-registered on a publicly available platform, and, as a result, the results should be considered exploratory.

## Results

**Figure 1.**
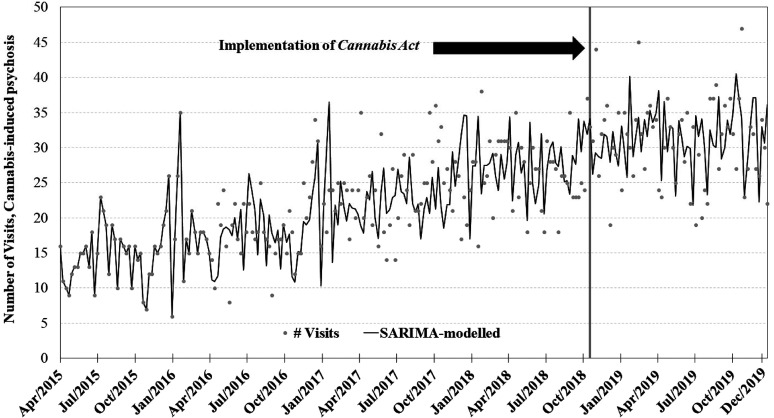
Weekly counts of conditions presenting to emergency departments in Ontario and Alberta before and after cannabis legalization, April 1, 2015–December 31, 2019.

**Figure 2.**
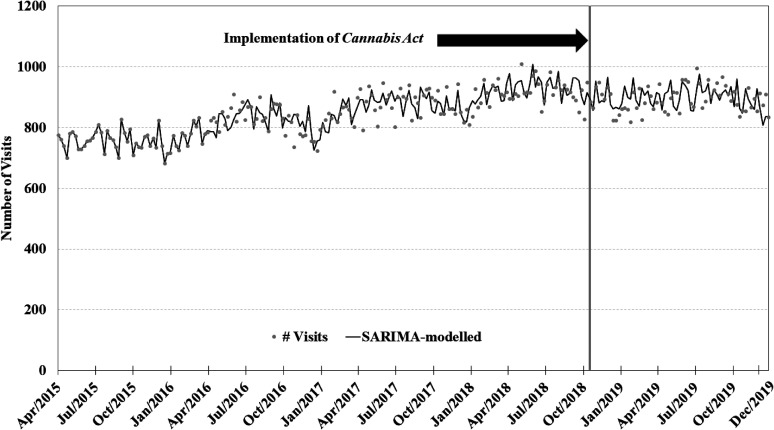
Weekly counts of schizophrenia conditions presenting to emergency departments in Ontario and Alberta before and after cannabis legalization, April 1, 2015–December 31, 2019.

**Figure 3.**
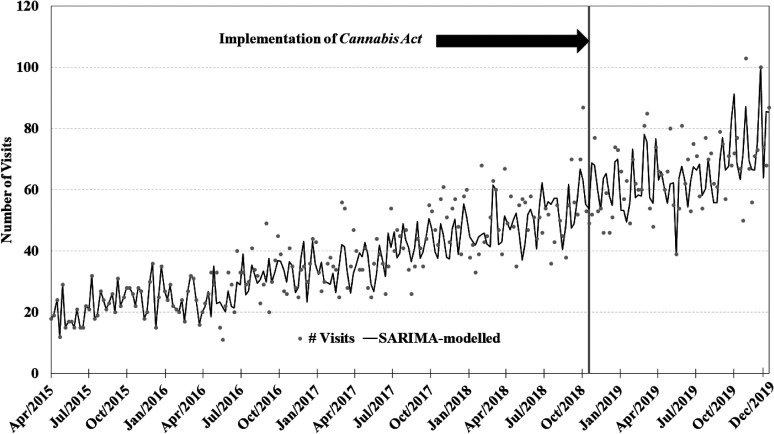
Weekly counts of amphetamine-induced psychosis conditions presenting to emergency departments in Ontario and Alberta before and after cannabis legalization, April 1, 2015–December 31, 2019.

**Figure 4.**
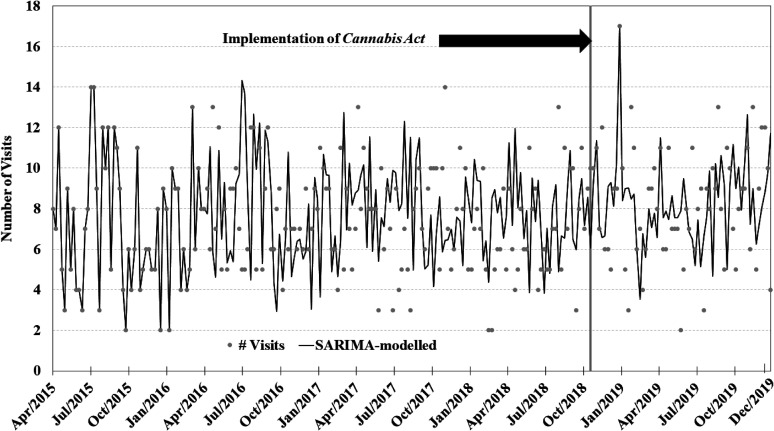
Weekly counts of alcohol-induced psychosis conditions presenting to emergency departments in Ontario and Alberta before and after cannabis legalization, April 1, 2015–December 31, 2019.

The visual depiction of the time series plots for each of the four primary SARIMA models can be found in Figures 1–4. Each SARIMA model worked upon 185 points pre-legalization and 63 points post-legalization.

Key parameters and results for all four SARIMA models can be found in Table 1. For the cannabis-induced-psychosis analysis, the SARIMA intervention term (assessing the step-function effect of cannabis legalization) was 0.34 (95% CI −4.1; 4.8; *P* = 0.88) visits per week, with the model showing no evidence of a step-function effect associated with cannabis legalization in relation to the cannabis-induced psychosis outcome (see Figure 1). There was one outlier identified in the final modeling (data point at week 240, with week 1 being the earliest calendar week), and this outlier point was accounted for in the final model. In model assessing weekly counts in the schizophrenia-and-related-conditions series, the non-significant step-effect intervention term was 24.34 (95% CI −18.3; 67.0; *P* = 0.26) presentations per week (see Figure 2). No outliers were detected. The SARIMA models working upon the amphetamine-induced psychosis series and the alcohol-induced psychosis series showed no evidence of significant step-effect changes associated with legalization: (1) step effect, amphetamine-induced psychosis, 1.93 visits per week (95% CI −2.8; 6.7; *P* = 0.43; 2 outliers identified at weeks 216 and 245; see Figure 3); (2) step effect, alcohol-induced psychosis, 0.61 visits per week (95% CI −0.6; 1.8; *P* = 0.31; 1 outlier identified at week 196; see Figure 4). It is important to note that the AIC values for these four primary step-function SARIMA models were higher than the series-specific models incorporating an attenuation parameter (which allows for the estimation of increases or decreases of the step function effect over time).

**Table 1.** Seasonal Autoregressive Integrated Moving Average (SARIMA) Models Assessing Associations Between Canada's Cannabis Legalization and Psychosis-Related Presentations to Emergency Departments in Ontario and Alberta, 2015–2019.^a^

SARIMA model	SARIMA notation	Local parameters		Seasonal parameters		Intervention parameter (visits) (95% CI)
		*P* (95% CI)	*q* (95% CI)	*P* (95% CI)	*q* (95% CI)	
Cannabis-induced psychosis	(0,1,2)(1,1,0)_52_	NA^b^	−0.87 (−1.0; −0.7)^*^ −0.11 (−0.2;0.0)	−0.36 (−0.5; −0.2)^*^	NA	0.34 (95% CI −4.1; 4.8), *P* = 0.88
Schizophrenia	(0,1,1)(1,1,0)_52_	NA	−0.84 (−0.9; −0.8)^*^	−0.42 (−0.6; -0.3)^*^	NA	24.3 (−18.3; 67.0) *P* = 0.26
Amphetamine-induced psychosis	(0,1,1)(1,1,0)_52_	NA	−0.97 (−1.0; −0.9)^*^	−0.42 (−0.6; −0.3)^*^	NA	1.93 (−2.8; 6.7) *P* = 0.43
Alcohol-induced psychosis	(0,1,1)(1,1,0)_52_	NA	−1.00 (−1.04; −0.96)^*^	−0.46 (−0.6; −0.3)^*^	NA	0.61 (−0.6; 1.8) *P* = 0.30

^a^ The results presented in this table are based on the combined Alberta and Ontario provincial data series; province-specific results were not estimated.

^b^ “NA” is “not applicable”: this parameter was not included in the final SARIMA model.

^*^ *P* < 0.001.

### Statistical Power Considerations

Given the null results related to our primary intervention effects across all of our four SARIMA models, we estimated the SARIMA step-function effect sizes that our designs would have had reasonable power (80%) to detect through the process of statistical power simulation (see Supplemental Table 1).^29^

## Discussion

The current study found no evidence that Canada's first phase of cannabis legalization was associated with significant changes over the 14.5-month post-legalization period in Ontario/Alberta emergency department presentations for either cannabis-induced psychosis or schizophrenia. Our time series models also showed no evidence of changes in our comparison series of alcohol-induced psychosis or amphetamine-induced psychosis. Given the lack of pre-registration of our study, our interpretation of results and discussion of key findings are exploratory.

Our null results appear similar to those described in a recent medical-chart-review study comparing the prevalence of pre- versus post-legalization psychotic-disorder presentations (*n* ≈ 741) occurring 2 years before and 5 months after legalization to two Sherbrooke, Québec psychiatric emergency department units, with the Sherbrooke-based study finding no evidence of any significant difference in the pre- versus post-legalization prevalence of a psychotic-disorder diagnosis in patients’ medical records.^30^ As the authors noted, however, the comparison of the prevalence of pre- versus post-legalization psychotic-disorder presentations may have been confounded by treatment-service changes occurring during the study period via the initiation of new community-based psychiatric-treatment outreach programs [i.e., new availability of intensive psychiatric follow-ups services in the community; creation of a specialized first-episode (FEP) psychosis team], and such alterations may have differentially affected patterns of psychotic-disorder presentations to the psychiatric ED study sites across the pre- and post-legalization periods.^30^ In relation to the current study, a number of factors may have contributed to the null results related to our target series. First, key indicators of patterns of cannabis use in Alberta and Ontario showed relatively little change after legalization. In Alberta, for example, while the prevalence of past-90-day cannabis did increase significantly between pre-legalization and post-legalization periods (16.4% vs. 19.3%), there was no evidence of post-legalization change in the prevalence among individuals who use cannabis daily/almost daily (7.2% vs. 7.1%)^31^—a pattern of use most closely linked to the likelihood of experiencing cannabis-related harms,^6,32^ including persistent psychotic syndromes.^8,31^ In Ontario, there was no evidence of pre- versus post-legalization changes in the prevalence of past-90-day cannabis use (15.5% vs. 17.5%) or daily/almost daily use among individuals who use cannabis (6.4% vs. 6.0%).^33^ Also, there was a long period of *de facto* decriminalization and normalization of cannabis use in Canada prior to legalization.^34^ As a result, the legalization of non-medical cannabis use may have had only a relatively small influence on patterns of cannabis use not only in the Ontario and Alberta general populations, but also in those subpopulations potentially at higher risk for developing psychotic conditions, such as adolescents and young adults, people with psychotic illnesses, or those with a family history of psychosis.

In addition, the implementation of the *Cannabis Act* may have produced—paradoxically—a decrease in the post-legalization access, availability, and potency of cannabis products in Ontario and Alberta through a variety of mechanisms: (1) in Ontario, for example, there were reports that local, unlicensed cannabis dispensaries closed during the period leading up to and shortly following legalization in order to qualify for licenses as legal cannabis retailers^35^; (2) government prices for licit cannabis were higher than those prices for corresponding black market products, which tended to have higher THC potencies (e.g., THC potency of dried-cannabis products: 20.5%, black market vs. 16.5%, regulated market)^35–37^; (3) the cannabis-legalization rollout faced supply-chain bottlenecks, leading to shortages and limited selection of cannabis products in government-licensed retail stores^38–41^; (4) while every province and territory had a government-sanctioned online retail cannabis store open at the time of legalization,^39^ there was a scarcity of brick-and-mortar cannabis stores open immediately following legalization, especially in Ontario [by March 2019, Alberta had opened 76 brick-and-mortar cannabis stores (the highest number of any province/territory), but Ontario had yet to open any brick-and-mortar cannabis stores^39^]; and (5) shortly after legalization, a national rotating postal strike occurred—an event which likely would have affected the delivery of cannabis products ordered on government-sanctioned online sales platforms.^42^

It is also possible that Canada's initial legalization of non-medical cannabis use occurring on October 17, 2018 (“Cannabis 1.0”)—and its concomitant legal restriction on the sale of high potency cannabis products—may have attenuated the potential impacts of legalization on the cannabis-induced-psychosis series and schizophrenia series in our paper. The *Cannabis Act* included a two-phased sequence regarding the availability of cannabis products, with industry-wide rules and standards regarding the types of cannabis products legally allowed for sale in licensed retail environments.^1^ During the first year of legalization (“Cannabis 1.0”), only dried-leaf cannabis products and oils were allowed to be sold in government-sanctioned retail outlets.^1,43^ In the second phase of legalization (“Cannabis 2.0”), cannabis edibles and high THC-potency cannabis concentrates (e.g., hashish), as well as high-potency cannabis vaping products, became legally available in licensed retail outlets in early 2020 in most provinces and territories.^1,43^ Recent research has shown that daily use of high-potency cannabis is significantly associated not only with the likelihood of developing a psychotic illness, but also that this likelihood is more pronounced among individuals residing in geographical regions which had greater availability of high potency cannabis products.^31^ Given the possible link between the daily usage and market availability of high-potency cannabis products and the development of psychotic illnesses, it will be important for future research to assess the impacts of the rollout of Canada's second phase cannabis legalization (“Cannabis 2.0”) on patterns of psychotic syndromes in Canadian provinces and territories.

Our results do show that ED presentations for cannabis-induced psychotic conditions increased approximately 2-fold during the study period, rising from approximately 15 episodes per week in April 2015 in the combined Ontario/Alberta data set to approximately 30 visits per week in December 2019 (see Figure 1). Similarly, national Canadian rates of inpatient hospitalizations with cannabis-induced psychotic conditions (ICD-10 F12.5) tripled from 2006 to 2016, increasing from 0.80 to 2.49 per 100,000 in the Canadian population.^44^ Prior international registry-based studies have found similar patterns: a recent study of all inpatient hospital psychiatric admissions in Denmark estimated that the admission rate for cannabis-induced psychosis approximately doubled from 2006 to 2016.^21^ The authors^21^ speculated that this increasing trend in cannabis-induced psychosis hospitalizations was associated with the parallel increases in the THC content of cannabis products in Denmark [(e.g., the THC potency of cannabis resin in Denmark increased from 13% THC in 2006 to 30% THC in 2016)^45^]. At this time, we are not aware of similar THC-potency indicators for cannabis products during the period of our study and, as a result, we are not able to evaluate the possible statistical association between indicators of THC potency across the study period and the observed cannabis-induced psychosis trend in our project. It is also important to note that amphetamine-induced psychosis presentations also increased substantially across the 2015–2019 period (see Figure 3). It may be important for future research to assess the possible mechanisms contributing to such increases in ED presentations for substance-induced psychotic disorders in Canadian settings.

The current study's findings need to be interpreted in light of its potential limitations. Given that our SARIMA design is a quasi-experimental approach, it is possible that unaccounted for variables may have confounded our assessment of the estimated relations between cannabis legalization and the outcome series. The study also relied on ICD-10 codes in ED electronic medical records to identify the target and comparison outcome series. We are not aware of studies assessing the validity of our ICD-10 classifications for the schizophrenia, cannabis-induced psychosis, alcohol-induced psychosis, or amphetamine-induced psychosis outcomes in our 2015–2019 Alberta or Ontario NACRS records in relation to well-validated diagnostic standards. Nonetheless, prior research has shown that a slightly broader ICD-10 classification of psychotic conditions including schizophrenia and other related syndromes (i.e., ICD-10 codes: F20.x, F22.x-F25.x, F28.x, F29.x, F30.2, F31.2, F31.5) drawn from Alberta hospital-based administrative medical records had substantial levels of accuracy and concordance vis-à-vis expert medical-chart-review diagnoses (positive predictive value = 90.4; kappa = 0.69).^46,47^ Our interrupted time-series approach chose to model the primary intervention as the date of legalization—October 17, 2018. While it may be possible to argue that our modeling approach should have included a lagged intervention date, we chose not to include a potential lagged effect for two reasons: (1) at least in our understanding, it is unclear what time period might constitute a more appropriate or defensible lag period, given the lack of scientific literature regarding the typical length of cannabis exposure required for exacerbation of pre-existing schizophrenia-related conditions or new-onset schizophrenia conditions vis-à-vis emergency department presentation; and (2) the current study incorporated a rather abbreviated 14.5-month post-legalization period, and the choice of, say, a 6-month or 12-month lag would make the follow-up even shorter and reduce the statistical power of the SARIMA models to detect any potential lagged effects. Future research may want to incorporate different research designs to account for a potentially longer latency period between legalization-associated changes in cannabis use patterns and emergent trends in persistent psychotic syndromes. We attempted to explain, to some extent, the null results in our study by pointing to national survey data showing no or minimal evidence of changes in the Ontario/Alberta prevalence of past-90-day cannabis use or daily/almost daily cannabis use. It is important to note that such prevalence estimates may not necessarily track neatly population-based harms, especially as these prevalence measures do not account for important aspects of patterns of use, such as the quantity of use, heavy episodic (binge) cannabis use, THC potency of cannabis products consumed, or typical route of administration—all of which may contribute unique components to patterns of population-based cannabis harms. It may be important for future research to incorporate more detailed assessments of cannabis-use patterns vis-à-vis legalization-associated changes in cannabis-related harms. As a result, the diagnostic validity of our outcome codes is uncertain. The primary ED-based ICD-10 outcome series of cannabis-induced psychosis and schizophrenia (and related disorders) do not include information capturing quantity, frequency, duration, or the age of initiation of cannabis use in the ICD-10 diagnostic categories. Also, our analyses combined Ontario and Alberta data to maximize statistical power considerations, but future research may want to assess how the different provincial rollout and retail strategies might be associated with cannabis-related and psychosis outcomes. Also, given the potentially idiosyncratic factors involved in the Canadian phased rollout of cannabis legalization, our results may not generalize to other settings.

Despite these limitations, we suggest that the study has a number of strengths, which support its contribution to the existing literature. Using population-based provincial ED records from Alberta and Ontario (the only two provinces in Canada with virtually 100% medical-record coverage of all emergency department presentations in their populations) and a quasi-experimental time-series design, the study provides a novel examination of the possible association between the implementation of cannabis legalization and population-based patterns of transient and persistent psychotic conditions—a central topic frequently discussed in reviews of the potential harms (and benefits) associated with legalization. In conclusion, the project showed no evidence of relations between Canada's first phase of cannabis legalization and ED presentations for cannabis-induced psychosis or schizophrenia-related conditions in the 14.5-month post-legalization period. Given that the *Cannabis Act* mandated a public review of the health benefits and harms associated with legalization, the current study aims not only to offer initial evidence for this Canadian evaluation, but also to provide preliminary support for similar discussions of the cost–benefit calculus in other jurisdictions. Future research will need to collect similar population-based cannabis-consumption and psychosis-outcome data over longer periods in different settings in order to reach firm conclusions regarding the potential impacts of cannabis legalization on transient and persistent psychotic syndromes in society.

### Data Access

The Canadian Institute of Health Information provided the study data to the lead authors (RCC, MS) under strict confidentiality and data-security constraints. As a result, the current study data cannot be released publicly with the manuscript. Nonetheless, study data are publicly available to qualified researchers through an application process to the Canadian Institute of Health Information. The lead author (RCC) can provide to qualified researchers details for supporting CIHI data requests for the data contained in this study.

## Supplemental Material

sj-docx-1-cpa-10.1177_07067437211070650 - Supplemental material for Associations Between Canada's Cannabis Legalization and Emergency Department Presentations for Transient Cannabis-Induced Psychosis and Schizophrenia Conditions: Ontario and Alberta, 2015–2019 Click here for additional data file. Supplemental material, sj-docx-1-cpa-10.1177_07067437211070650 for Associations Between Canada's Cannabis Legalization and Emergency Department Presentations for Transient Cannabis-Induced Psychosis and Schizophrenia Conditions: Ontario and Alberta, 2015–2019 by Russell C. Callaghan, Marcos Sanches, Robin M. Murray, Sarah Konefal, Bridget Maloney-Hall and Stephen J. Kish in The Canadian Journal of Psychiatry
