# Supplementary material for: Associations Between Canada's Cannabis Legalization and Emergency Department Presentations for Transient Cannabis-Induced Psychosis and Schizophrenia Conditions: Ontario and Alberta, 2015–2019
Source: Can J Psychiatry. 2022 Jan 12;67(8):616–25. doi: 10.1177/07067437211070650 (PMC9301152; doi:10.1177/07067437211070650)
Supplement: sj-docx-1-cpa-10.1177_07067437211070650 - Supplemental material for Associations Between Canada's Cannabis Legalization and Emergency Department Presentations for Transient Cannabis-Induced Psychosis and Schizophrenia Conditions: Ontario and Alberta, 2015–2019 [file sj-docx-1-cpa-10.1177_07067437211070650.docx]

**Supplemental Table 1.** Statistical power simulations vis-a-vis the primary four SARIMA models.

| SARIMA Model^a^ | Total visits in series | Model estimated number of ED visits at legalization date | σ  (standard deviation of pre-intervention series) | Detectable effect (ω), as number of  outcome ED visits | Effect,  % change | Scaled effect δ, with δ = ω/σ. |
| --- | --- | --- | --- | --- | --- | --- |
|  |  |  |  | At 80% Power | At 80% Power | At 80% Power^b^ |
|  |  |  |  |  |  |  |
| Cannabis-induced | 5832 | 31.9 | 6.35 | 3.7 | 12% | 0.58 |
| Schizophrenia | 211,682 | 920.2 | 40.42 | 44.2 | 5% | 1.09 |
| Amphetamine-induced | 10,831 | 56.8 | 8.64 | 7 | 12% | 0.81 |
| Alcohol-induced | 1884 | 8.96 | 3.51 | 2.2 | 25% | 0.63 |

^a^ The SARIMA models working upon the synthetic (simulated) data seta had the same local and seasonal SARIMA parameters (*p*, *d*, *q*) and similar lag 1 autocorrelations and lag 52 autocorrelations as the SARIMA models working upon the original data (described in Table 1 of the manuscript). We used the pre-intervention series to simulate a full data series, including both the pre-legalization and post-legalization periods. Then we added a step effect to the post-legalization series by adding a constant to all points after legalization. Next, we used the SARIMA analytic model to see if the model detected the step-function effect as statistically significant (at alpha level 0.05) – and how frequently the detection occurred. For example, if the SARIMA model detected the step-function effect 80% of the time, the empirically derived statistical power would be equal to 0.80 for the tested effect size, given the input parameters. We included 5 to 10 effects per model (in order to calibrate the effect to correspond to a statistical power of 0.80), and we ran 100 replications for each effect size in order to estimate the power curve around the effect size for which the power was 80% (the resulting figures are available from the first author). ^b^ These effect sizes are expressed as a scaled effect δ, with δ = ω/σ, where σ is the standard deviation of the pre-intervention time series and ω is the size of the unscaled effect (expressed as number of target outcome presentations) associated with cannabis legalization.
